# Ilixadencel – an Allogeneic Cell-Based Anticancer Immune Primer for Intratumoral Administration

**DOI:** 10.1007/s11095-018-2438-x

**Published:** 2018-06-14

**Authors:** Alex Karlsson-Parra, Juliana Kovacka, Emilia Heimann, Margareth Jorvid, Sijme Zeilemaker, Sharon Longhurst, Peter Suenaert

**Affiliations:** 1 Immunicum AB, Grafiska Vägen 2, 412 63 Gothenburg, Sweden; 2 Department of Immunology, Genetics and Pathology, Rudbeck Laboratory, Uppsala University, Dag Hammarskjölds Väg 20, 752 37 Uppsala, Sweden

**Keywords:** cross-presentation, dendritic cell, intratumoral, ilixadencel, NK cell

## Abstract

Intratumoral administration of an immune primer is a therapeutic vaccine strategy aimed to trigger dendritic cell (DC)-mediated cross-presentation of cell-associated tumor antigens to cytotoxic CD8^+^ T cells without the need for tumor antigen characterization. The prevailing view is that these cross-presenting DCs have to be directly activated by pathogen-associated molecular patterns (PAMPS), including Toll-like receptor ligands or live microbial agents like oncolytic viruses. Emerging data are however challenging this view, indicating that the cross-presenting machinery in DCs is suboptimally activated by direct PAMP recognition, and that endogenous inflammatory factors are the main drivers of DC-mediated cross-presentation within the tumor. Here we present preclinical mode of action data, CMC and regulatory data, as well as initial clinical data on ilixadencel. This cell-based drug product is an off-the-shelf immune primer, consisting of pro-inflammatory allogeneic DCs secreting high amounts of pro-inflammatory chemokines and cytokines at the time of intratumoral administration. The mechanism of action of ilixadencel is to induce recruitment and activation of endogenous immune cells, including NK cells that subsequently promotes cross-presentation of cell-associated tumor antigens by co-recruited DCs.

## Introduction

Agents known as immune check point inhibitors (CPIs), particularly therapeutic antibodies that block the immunosuppressive programmed cell death protein-1 (PD-1) and the programmed cell death-ligand 1 (PD-L1) pathway, are now revolutionizing the practice of medical oncology due to their ability to improve outcomes in various malignancies. In contrast, with the exception of some forms of premalignant disease, the proportion of patients benefiting from treatment with cancer vaccines leaves much to be desired [1].

In most mouse models, anti-tumor immunity requires the generation of tumor-specific cytotoxic CD8^+^ T cells (CTLs) and it is similarly believed that CTLs are major players in successful immunotherapies of human cancers. There is now emerging evidence that PD-1/PD-L1 inhibitors are more effective in tumors that are already recognized by the immune system, as manifested by a pre-existing CD8^+^ T-cell infiltrate [2], indicating that anti-PD-1/PD-L1 antibodies reactivate disabled intratumoral T cells [3]. In patients with no pre-existing antitumor T cells, a vaccine-induced reactivation of tumor-specific T cells and also activation of T cells from the naive repertoire is therefore a rational option to counteract clinical resistance to CPIs [4]. Indeed, recent findings indicate that the lack of spontaneous immune infiltration in solid tumors is unlikely due to a lack of tumor-specific antigens that potentially may be recognized by the immune system [5]. The vaccine approach could thus potentially transform a non-inflamed” cold” non-permissive tumor resistant to CPIs into a sensitive inflamed” hot” tumor [6]. Combining cancer vaccines with CPIs or other anti-cancer drugs, like gemcitabine and sunitinib, known to counteract myeloid-derived suppressor cell (MDSC)-derived immunosuppression [7] may thus provide the necessary stimulation to broaden the repertoire of T cells engaged in the antitumor response.

### Mutation-Derived Tumor Antigens (Neoantigens)

Effective anti-tumor immunity induced by CPIs or adoptive T cell therapy in humans has been associated with the presence of T cells directed at cancer neoantigens [8, 9], a class of human leukocyte antigen (HLA)-bound peptides that arise from tumor-specific mutations [10, 11]. These mutation-derived and patient-specific neoantigens are usually highly immunogenic because they are not present in normal tissues and hence bypass central thymic tolerance [12]. These facts have breathed new life into the field of cancer vaccines, and treatment with neoantigen-based vaccines, in which the patient’s neoantigens are first characterized and then synthesized *in vitro*, is presently undergoing several clinical trials and initial clinical data are indeed promising [13, 14].

However, on a purely practical level, the manufacturing process of neoantigen-based vaccines includes many obstacles that will need to be overcome. In addition, this production is entirely patient dependent, i.e. can only be performed after the neoantigens for each individual patient have been characterized from a tissue sample taken from the patient’s own tumor which constitutes quite a logistical challenge [10, 11].

### Intratumoral (*In Situ*) Administration of Immune Primers

A rational way to circumvent practical problems associated with characterization and *in vitro* production of tumor neoantigens is to use the patient’s existing tumor (or metastasis of) as a direct neoantigen source by injecting an immune primer directly into the patient’s own tumor. Such an approach would allow for the development of vaccines in patients themselves, thereby minimizing the resource allocation required in ex vivo processing. Furthermore, this strategy may take advantage of the complete neoantigen repertoire of the patients tumor rather than be limited to a restricted number of characterized and *ex-vivo* produced tumor neoantigens [15].

### The Immunosuppressive Tumor Microenvironment

The tumor microenvironment (TME) contains stromal cells and immune cells that shape cancer development and impact the response to tumor therapy [16]. Intratumoral immune cells comprise lymphocytes, such as T cells, and natural killer (NK) cells, and diverse populations of myeloid cells, including MDSC, macrophages, and dendritic cells (DCs) [16]. Simplistically, intratumoral MDSCs, M2-polarized macrophages and regulatory CD4^+^ T cells (Treg) can promote cancer cell growth, angiogenesis, and metastasis, as well as contribute to the establishment of an immunosuppressive environment. The presence of these cells within the tumor is associated with tumor progression and poor clinical outcome [17]. Additionally, tumor stromal fibroblasts have recently been shown to be major producers of immunosuppressive TGF-β that inhibits T cell recruitment into the tumor [18, 19], thus potentially explaining why certain tumors with a high mutational load still lack infiltrating T cells [20].

### Conventional Type 1 DCs

It is well understood that antigen-presenting cells within tumors typically do not maintain cytotoxic CD8^+^ T cell (CTL) function, despite engaging them. Across multiple mouse tumor models and human tumor biopsies, intratumoral conventional type 1 DCs (cDC1), bearing CD103 in mouse and CD141 in humans, are extremely sparse and yet remarkably capable stimulators of CTLs [21, 22]. These are uniquely dependent upon Batf3 transcription factors and generated by GM-CSF and Flt3L cytokines. Regressing tumors have higher proportions of these cells, T-cell dependent immune clearance relies upon them, and abundance of their transcripts in human tumors correlates with clinical outcome [21, 22]. The cDC1 subset is especially adapted at taking up cell-associated antigens from dying tumor cells and transporting tumor-derived antigens to tumor-draining lymph nodes where they constitute the key DC subtype responsible for cross-presentation of tumor-derived antigens to tumor-specific CD8^+^ T cells [22, 23]. In addition to this trafficking role, cDC1 also play a key role within tumors themselves by re-stimulating and expanding tumor-specific CD8^+^ T cells [21], and support T cell effector function by secreting interleukin (IL)-12p70 [24]. The overall importance of cDC1 in anti-tumor immunity is underscored by multiple studies demonstrating that the lack of cDC1 in Batf3 knock out mice abolishes the rejection of immunogenic tumors and the response to adoptive T cell therapy and to immune checkpoint blockade [21, 22].

### Recruitment of DCs

Since cDC1s are usually very sparse within the tumor, therapies aimed at increasing intratumoral cDC1 abundance are expected to boost anti-tumor immunity and potentially increase the responsiveness of cancer patients to immunotherapy inhibiting tumor-derived immunosuppression [21, 22]. Recently, a key role for intratumoral NK cells was uncovered by their production of chemoattractants, including the chemokine RANTES (also known as CCL5), that are necessary for the accumulation of cDC1 in incipient tumors and for tumor immune control in mouse models [25]. Evidence were further provided that a similar NK cell/ chemokine functional axis determines cDC1 abundance in human melanoma, breast cancer, lung cancer, and head and neck squamous cell carcinoma and show that it impacts on patient survival [25].

### Induction of Th1-Polarizing Mature DCs

Different types of immune primer, including different Toll-like receptor (TLR) ligands and pro-inflammatory cytokines, including TNF-α and IL-1β, are well-known DC activators. One issue that remains to be fully addressed is the choice of primer(s) that would appropriately stimulate both DC-mediated T-helper 1 (Th1) polarization of tumor-specific CD4^+^ T cell and cytotoxic CD8^+^ T cell (CTL) responses. Activated/mature DCs are characterized by their expression of membrane-bound co-stimulatory molecules like CD80 and CD86 and may potentially secrete the Th1- and CTL-polarizing factor IL-12p70. The ability to secrete IL-12p70 is, however, not an intrinsic attribute of activated DCs and uncommitted immature DC thus require concomitant exposure to IFN-γ when activated by TLR ligands and/or pro-inflammatory cytokines, to develop the capacity to produce high levels of IL-12p70 upon subsequent contact with helper T cells expressing the CD40 ligand. This effect is specific for IFN-γ and is not shared by other IL-12-inducing factors [26].

### Cross-Presentation

The ability to cross present soluble or cell-associated tumor antigens to tumor-specific CD8^+^ T cells is key to the antigen-presenting function of mature tumor antigen-loaded DCs. As mentioned earlier, conditions of DC maturation have been shown to be important for DCs ability to activate T cells by the expression of co-stimulatory molecules and production of the Th1-deviating cytokine IL-12p70. However, it remains partially unknown how different pathways of DC maturation are associated with modulation of their ability to cross present captured exogenous cell-associated tumor antigens. When a minimal peptide epitope is used for antigen-loading of DCs, no additional processing of the peptide is required to generate HLA class I antigen-peptide complexes. In contrast, the generation of such complexes from soluble or cell-associated proteins released from dying tumor cells requires phagocytosis/endocytosis and processing by the antigen-processing machinery. The cross-presenting pathway requires antigen export of polypeptides from endosomal compartments into the cytosol, proteasomal digestion and transport by the transporter associated with antigen processing (TAP)-subunits TAP1 and TAP2 of polypeptides to the endoplasmic reticulum or endosomes, where final peptide trimming and MHC-I peptide loading take place [27, 28]. Notably, recent studies indicate that activated NK-cells, by their combined production of IFN-γ and TNF-α, efficiently induce cross-presentation of cell-associated tumor antigens in human monocyte-derived DCs [29–31] and human CD141^+^ cDC1 [29], the latter observation highlighting a previously unknown cross-talk between NK cells and CD141^+^ DCs for inducing tumor cell-derived antigen cross-presentation. In comparative in vitro studies using monocyte-derived DCs (MoDC) [29], it was demonstrated that the concomitant release of IFN-γ and TNF-α from activated NK-cells was clearly superior to direct stimulation with TLR3 and TLR7/8 ligands in promoting cross-presentation of cell-associated tumor antigens by monocyte-derived DCs. Stimulation with IFN-γ plus TNF-α was also highly efficient in promoting cross-presentation of cell-associated tumor antigens by human cDC1 (CD141^+^ DCs). The poor or modest induction of cross-presentation in MoDCs by the TLR ligand poly I:C (TLR3 ligand) and R848 (TLR7/8 ligand) is in line with other reports from *in vitro* studies using MoDCs where single-stimulation with poly I:C (or IFN-α) did not affect TAP-expression in MoDCs while single-stimulation with IFN-γ induced significantly increased expression of both TAP-1 and TAP-2 [32].

At face value, these findings are contradicted by multiple reports from mouse models, indicating that intratumoral injection of TLR ligands like poly I:C is inducing subsequent priming of CD8^+^ T cells and also challenges the current” dogma” that a DC must become directly activated through pattern recognition receptors, including TLRs, in order to efficiently induce a CTL response. Reis e Sousa and colleagues [33] thus reported that APCs indirectly activated by pro-inflammatory endogenous factors have the capacity to promote the proliferation of naïve CD8^+^ T cells but that this does not lead to sustained clonal expansion and does not suffice to generate effector T cells endowed with CTL properties. However, since the model tumor antigens that were used in these studies were minimal peptide epitopes, the validity of these observations for cell-associated tumor antigens which have to be cross presented, was not addressed.

Taken together, an intratumorally injected immune primer should ideally be able to induce recruitment of cDC1 and induce tumor cell death facilitating the release of cell-associated neoantigens for subsequent capture by recruited DCs [22]. The immune primer should moreover be able to promote DC-mediated cross-presentation of captured cell-associated neoantigens, as well as induce proper DC-activation, in order to induce systemic Th1-deviated anti-tumor T cell responses, allowing the neoantigen-specific T cells to attack and destroy the treated tumor and also distant tumor lesions [34]. Finally, an optimal immune primer would preferably also inhibit additional intratumoral immunosuppressive pathways that are not targeted by co-delivered immuno-suppression blockers like CPIs.

### Intratumorally Injected Allogeneic Pro-Inflammatory DCs as” off-the-shelf” Anticancer Immune Primers

As to potential potent immune enhancers that can be used for intratumoral administration, recent studies have shown that autologous DCs, which have been pre-activated during a limited time period *ex vivo*, have the potential to indirectly prime naïve CD8^+^ T cells *in vivo*, by acting as a pure immunogenic adjuvant/primer affecting recruited endogenous DCs and not as antigen presenters [35, 36]. This indirect immune enhancing function of DCs was strictly dependent on their active secretion of certain DC- and NK-cell-recruiting chemokines at the time of administration [35]. In line with these data, pre-clinical findings indicate that the efficient induction of antigen-specific CTLs, characterizing viral infections, is caused by cross-priming, where initially infected DCs produce a unique set of inflammatory factors that recruit and activate non-infected “bystander” DCs [37–39]. Notably, in the study by Pang *et al*., signaling through the interleukin 1 receptor (IL-1R) was required for productive cross-presentation of cell-associated viral antigens to CD8^+^ T cells, while signaling through the pattern recognition receptors TLR7 and RIG-I was not. In line with these findings, a recent study showed that cross-presentation of cell-associated viral antigens by human CD141^+^ cDC1, which are constitutively resistant to viral infection, rely on viral antigens produced in infected bystander DCs [40]. By dissociating viral infection from antigen presentation, this mechanism protects the functional capacity of cDC1 to launch a CTL-mediated adaptive immunity against viral infection.

*In vitro* studies, using human MoDCs, have further shown that these cells can be programmed to produce vigorous amounts of Th1-associated chemokines and cytokines in a sustained fashion when stimulated with a combination of stimulatory factors, including Toll-like receptor (TLR) ligands and IFN-γ, thus mimicking virally infected DCs [41, 42]. Notably, activated MoDCs were found to induce recruitment of NK cells *in vitro* [42]. When comparing the ability to recruit immunosuppressive FOXP3^+^ regulatory T cells (Treg) between different DC-types used in the clinical setting, PGE2 matured DCs, but not type-1 polarized DCs (generated in the presence of the TLR3 ligand poly I:C plus IL-1β, TNF-α, IFN-γ and IFN-α), showed high Treg-attracting activity [43]. Moreover, in the study by Pang *et al*., using transgenic mice, it was shown that no MHC compatibility was needed between infected DCs and non-infected, cross-presenting, DCs [39]. By using allogeneic DCs as immune primers in the vaccine setting, such cells will further be regarded as foreign allogeneic invaders that most likely will potentiate a Th1-deviated inflammatory reaction, further promoting recruitment and activation of endogenous” bystander” DCs at the vaccination site [44].

## Studies with Pro-Inflammatory Allogeneic Dendritic Cells

### Preclinical Studies *In Vitro*

In a recently published paper [45], the interaction of pro-inflammatory DCs, activated with a highly potent cocktail consisting of poly I:C (TLR3 ligand), R848 (TLR7/8 ligand) and IFN-γ [41] with allogeneic peripheral blood mononuclear cells (PBMCs) (mimicking the interaction of intratumorally injected allogeneic DCs and recruited immune cells from the patient) was investigated. To summarize, pro-inflammatory DCs were found to produce several Th1-associated cytokines and chemokines in a sustained fashion, including IL-12p70 and CXCL-10 (IP-10). The production of pro-inflammatory factors by GMP-produced pro-inflammatory DCs (ilixadencel; see manufacturing process below) stimulated with the same cocktail have further been shown to also produce TNF-α, IL-1β and MIP-1α as well as high amounts of IL-12p70 and RANTES [46]. Moreover, this type of pro-inflammatory DCs were found to efficiently activate co-cultured allogeneic NK cells and T cells and also supported strong cytolytic function of NK-cells. The interaction with allogeneic PBMCs further created a milieu in favor of maturation of immature” bystander” DCs. Finally, when a cell-associated antigen was provided exogenously, supernatants from co-cultures with these pro-inflammatory DCs and allogeneic PBMCs promoted a highly efficient cross-presentation in bystander MoDCs (more than 10-fold increase when compared to non-treated immature MoDCs) of cell-associated tumor antigens to antigen-specific, IFN-γ producing CD8^+^ T cells [45].

### Preclinical Studies *In Vivo*

In* in vivo* mouse models, intratumoral administration of allogeneic mouse DC activated with an identical stimulation cocktail as used for human DCs (except that human recombinant IFN-γ was replaced by the mouse equivalent) was shown to induce recruitment of NK cells and T cells into the tumor while the numbers of MDSCs and regulatory T cells decreased [47]. When injected subcutaneously, these allogeneic DCs were shown to initiate host DC activation and migration to draining lymph node, leading to T cell activation. Injection of pro-inflammatory allogeneic DCs into a site (subcutaneous tissue or melanoma tumor) transfected with a virus vector, inducing local expression of the tumor antigen gp-100, resulted in the generation of gp-100 specific CD8^+^ T cells and when combined with transfer of gp100-specific pmel-1 T cells, a prolonged survival of B16-F10 melanoma-bearing mice was observed [47].

**Fig. 1:**
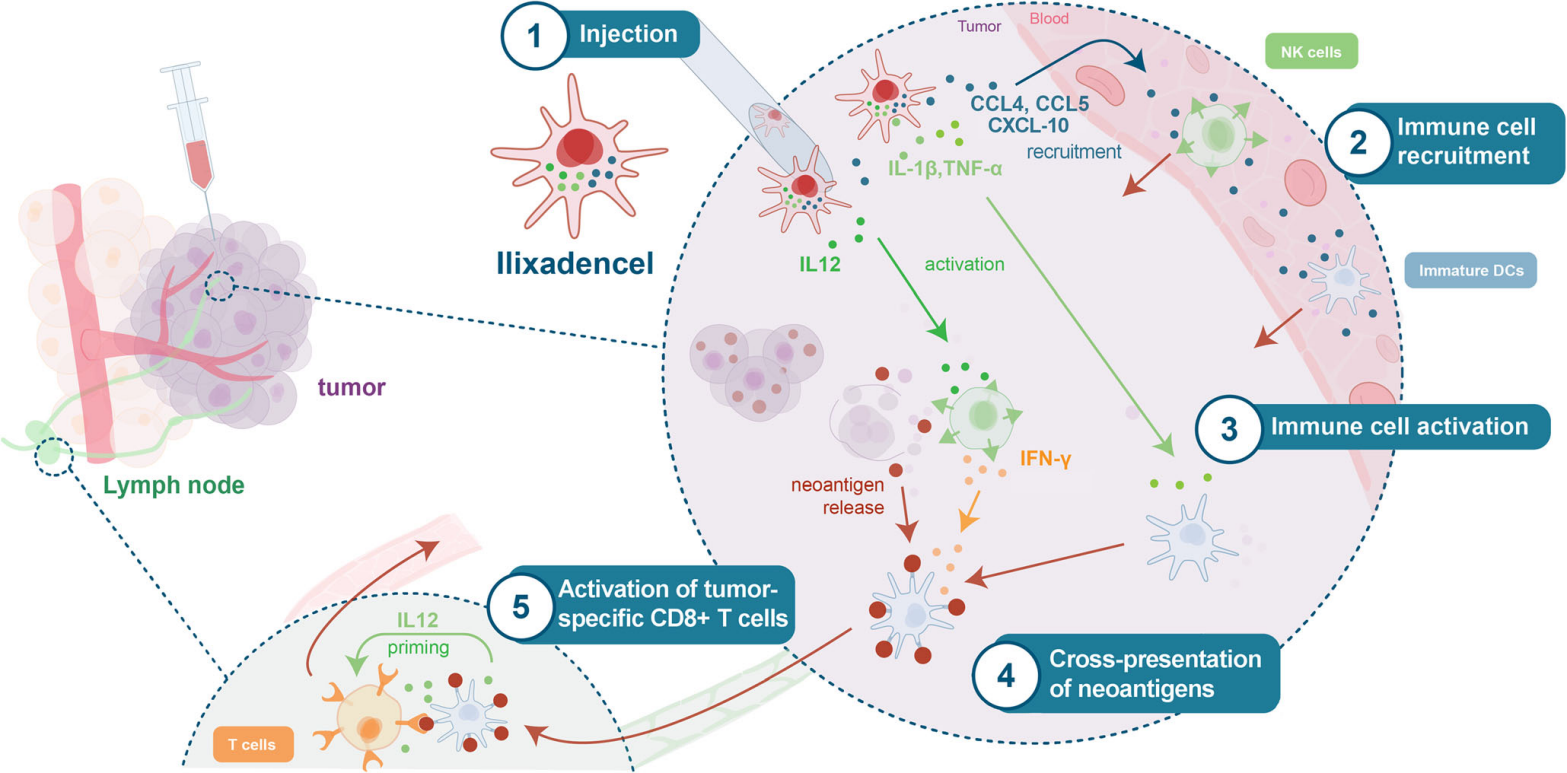
**Proposed mode of action for intratumorally injected ilixadencel.** After administration into the viable part of the tumor, ilixadencel DCs will release Th1-associated chemokines, including CCL4, 5 and CXCL10 that recruit immune cells, including NK-cells and pre-DCs into the tumor. The interaction between recruited NK-cells and ilixadencel DCs induce NK-cell-mediated killing of adjacent tumor cells, resulting in release of tumor-associated antigens, including neoantigens. The production of IFN-γ by activated NK-cells and TNF-α/IL-1β released by ilixadencel DCs will induce maturation as well as increase cross-presentation of engulfed cell-associated tumor antigens by recruited endogenous “bystander” DCs. Migration of these antigen-loaded and matured “bystander” DCs to the tumor-draining lymph node will finally lead to Th1-polarized activation of tumor-specific T cells, including cytotoxic CD8^+^ T cells, by the release of IL-12.

Based on these findings, we therefore expect that intratumorally injected pro-inflammatory allogeneic DCs (ilixadencel) in the clinical setting will induce recruitment of immune cells including NK-cells, DCs and T cells to the injection site. The cross-talk between the DCs and recruited NK cells will induce NK cell activation, subsequently leading to local tumor cell killing and release of cell-associated tumor antigens. NK-cell derived IFN-γ, in concert with TNF-α produced by the injected DCs and also by activated NK cells, will enhance cross-presentation of captured tumor antigens by recruited, endogenous, DCs. These antigen-loaded and cross-presenting DCs will start to mature due to activation by pro-inflammatory factors like TNF-α and IL-1β released by the injected allogeneic DCs. Production of IFN-γ by recruited and subsequently activated NK cells and alloreactive T cells will furthermore favor the differentiation of Th1 polarizing DCs [44]. Additionally, NK-cell and alloreactive T cell-derived IFN-γ may inhibit immunosuppressive M2-macrophages [48] and drive Treg fragility within the tumor [49]. The proposed mode of action for intratumorally injected ilixadencel is illustrated in Fig. 1.

### Toxicology Studies

Intrarenal injection of allogeneic rat pro-inflammatory DCs (surrogate rat product) did not cause any severe changes in the clinical chemistry parameters investigated in the toxicology studies. Histological examination of major organs did not indicate any tissue damage or local accumulation of inflammatory cells (ectopic foci). Hematological parameters (mirroring bone marrow function) did not indicate bone marrow toxicity. These data indicated that no organ-specific autoimmune reactions developed and that potential seeding of cells from the administration site to remote organs did not induce local foci of inflammatory cell immune reactions that would reflect areas of *in situ* mixed leukocyte reaction induced by seeded viable DCs.

### Biodistribution Studies

Radiolabelled (^111^In) pro-inflammatory rat DCs (surrogate rat product) were injected intrarenally into a rat strain that is allogeneic to the DCs. No significant radioactivity signals were detected in the organs with a physiological barrier, such as the brain, gonads, skin and muscles. For the signals observed up to 72 h after administration in lungs, liver, spleen, bone marrow and kidneys (injected kidney as well as contralateral kidney), it was not evident if signals came from free or cell bound ^111^In. With the knowledge that injected DCs are not viable for more than 2 to 3 days after injection, and that histology of major organs did not indicate any tissue damage or local accumulation of inflammatory cells (ectopic foci), the signals detected in different organs, most likely distributed through passive transport by the bloodstream, might reflect ^111^In-labelled dead cells or cell debris.

## Ilixadencel Drug Product

The active substance ilixadencel (INN) is composed of monocyte-derived dendritic cells (MoDCs) that have been stimulated with a combination of activating factors (R848, Poly I:C and IFN-γ) *ex vivo*, resulting in a residual production of desirable pro-inflammatory factors (such as TNF-α, IL-1β and MIP-1β and also high amounts of IL-12p70 and RANTES) and expression of co-stimulatory surface molecules (such as CD86) at the time of administration [46].

### Manufacturing

The drug product is manufactured using a continuous process, starting with monocytes enriched from leukapheresis source material from a healthy donor and ending with a cryopreserved drug product, formulated in human plasma and DMSO (10% (*v*/v)) at a concentration of 11.7 × 10^6^ viable cells/mL. Drug product is filled into 2 mL polyolefin vials with chlorobutyl, Flurotec® coated, stoppers and aluminum crimp caps. It is stored in vapor phase nitrogen (≤ −130°C) and is ready to use directly after thawing with no processing beyond extraction from the cells from the vial. The current shelf life of the product is 36 months when stored under real-time storage conditions (vapor phase nitrogen at ≤ −130°C).

Using leukapheresis material collected from one donor, one batch of ilixadencel is sufficient to treat 25–50 patients with a two-dose regimen.

### Adventitious Agents Safety Evaluation

The guidelines for CBMP (EMEA/CHMP/410869/2006, section 4.2.1) are followed, meaning that stringent sourcing requirements and acceptance criteria are in place for all raw materials and excipients derived from human sources. Donor screening and eligibility criteria are applied in accordance with country-specific law, EU directives (2006/17/EC and 2004/23/EC) and US requirements (CFR 1271.75). Additional testing is applied in accordance with specific member state regulatory requirements. The risk of BSE/TSE transmission is considered negligible due to the use of permanent deferral criteria for donors, and the fact that no materials of bovine origin are used in the manufacturing process.

## Regulatory Activities

In December 2015, WHO published, for the first time, a guideline on how to establish an International Nonproprietary Name (INN) for Cell Therapy Products (CTP) (*INN Working Doc. 13.323 revision 4*). The randomly chosen prefix (ilixa-) was to be followed by an infix describing cell type (−den-), for dendritic cells, and ended by the suffix (−cel) for all cell therapy products. To the best of our knowledge, Immunicum was one of the first companies that applied for an INN for a cell therapy product, and received in January 2017 the proposed recommended name ilixadencel (INN) from WHO that was later confirmed (*WHO Drug Information, Vol. 31, No. 3, 2017 Recommended INN: List 78)*.

Regulation (EC) No 1394/2007 introduced a new type of product classification in Europe, Advanced Therapy Medicinal Products (ATMP). This regulation gave for the first time in Europe, clarity on how to classify cell- and gene therapy products and tissue engineered products. The European Medicines Agency (EMA) established a special committee, the Committee for Advanced Therapies (CAT), to be involved in classification of, scientific advice for and review of these products. Immunicum applied for, and received in December 2015, classification by CAT/EMA for ilixadencel, as a somatic cell therapy medicinal product under Regulation 1394/2007. In the US, cell therapy products are handled and reviewed by the Center for Biologics Evaluation and Research (CBER) division at the US Food and Drug Administration (FDA).

During development of any medicinal product, and in particular for cell therapy products being a relatively new group of therapies to treat patients, early interactions with regulatory authorities are very important. Both national authorities in Europe, EMA and FDA have established procedures for scientific advice and meetings during development. In the US, during the pre-submission stage and in dialogue with the FDA, Immunicum received guidance on the development plan, and the data and regulatory requirements for the Investigational New Drug (IND) submission. This was important for a successful IND submission and the IND clearance by FDA in December 2016 made it possible for the company to include US patients in the IM-201 (MERECA) study. Immunicum has also received EMA scientific advice and recently national advice from several regulatory authorities in EU on the development and design of study IM-202.

The European Medicines Agency’s Committee for Advanced Therapies (CAT) provides a certification procedure for ATMPs under development by micro, small and medium-sized enterprises (SMEs). This is an opportunity for SMEs to receive an assessment of the data generated at that stage in development, to check that they are on the right track for successful product development. The certification procedure involves the scientific evaluation of quality data and, when available, non-clinical data that SMEs have generated at any stage of the ATMP development process. Immunicum submitted data under this procedure to EMA and received in March 2018 the ATMP Certificate for Quality and Nonclinical Data for ilixadencel.

## Clinical Studies

To date, two early clinical trials have been completed and two trials are currently active, as shown in Table I. The first-in-human phase I open-label study was completed in 2014 and evaluated safety and immunologic response of ilixadencel in patients with newly diagnosed metastatic renal cell carcinoma (mRCC) (IM-101). The phase I open-label study, IM-102, tested ilixadencel alone and in combination with sorafenib in patients with advanced hepatocellular carcinoma (HCC). Recruitment is now complete in both the phase I study in gastrointestinal stromal tumors (GIST) (IM-103) and the multicenter comparative phase II study in patients with newly diagnosed mRCC (IM-201).

**Table I:** Overview of the Clinical Trials with Ilixadencel

Study No	ClinicalTrials.gov identifier	Phase	Indication	Site(s)	Patients planned	Status
IM-101	NCT01525017	I	mRCC	Uppsala University Hospital, Sweden	12	Completed
IM-102	NCT01974661	I	HCC	Sahlgrenska University Hospital, Sweden	18	Completed
IM-201	NCT02432846	II	mRCC	Multicenter in Europe and US	88	Active, and recruitment completed
IM-103	NCT02686944	I	GIST	Karolinska University Hospital, Sweden	Appr. 12	Active, and recruitment completed

Overall, the safety profile observed across all trials is very good and is characterized by predominantly fever and chills as the treatment-related adverse events, and no DLT was found in doses up to 20 × 10^6^ viable cells. Likewise, no evidence of autoimmunity or clinically significant allo-immunity was found in any trial. On the other hand, in both studies there was evidence of a tumor-specific immune response following treatment with ilixadencel. Finally, signs of antitumor activity were observed among patients with mRCC and advanced HCC in the completed trials.

### Phase I Study in Patients with Newly Diagnosed Metastatic RCC

**Fig. 2:**
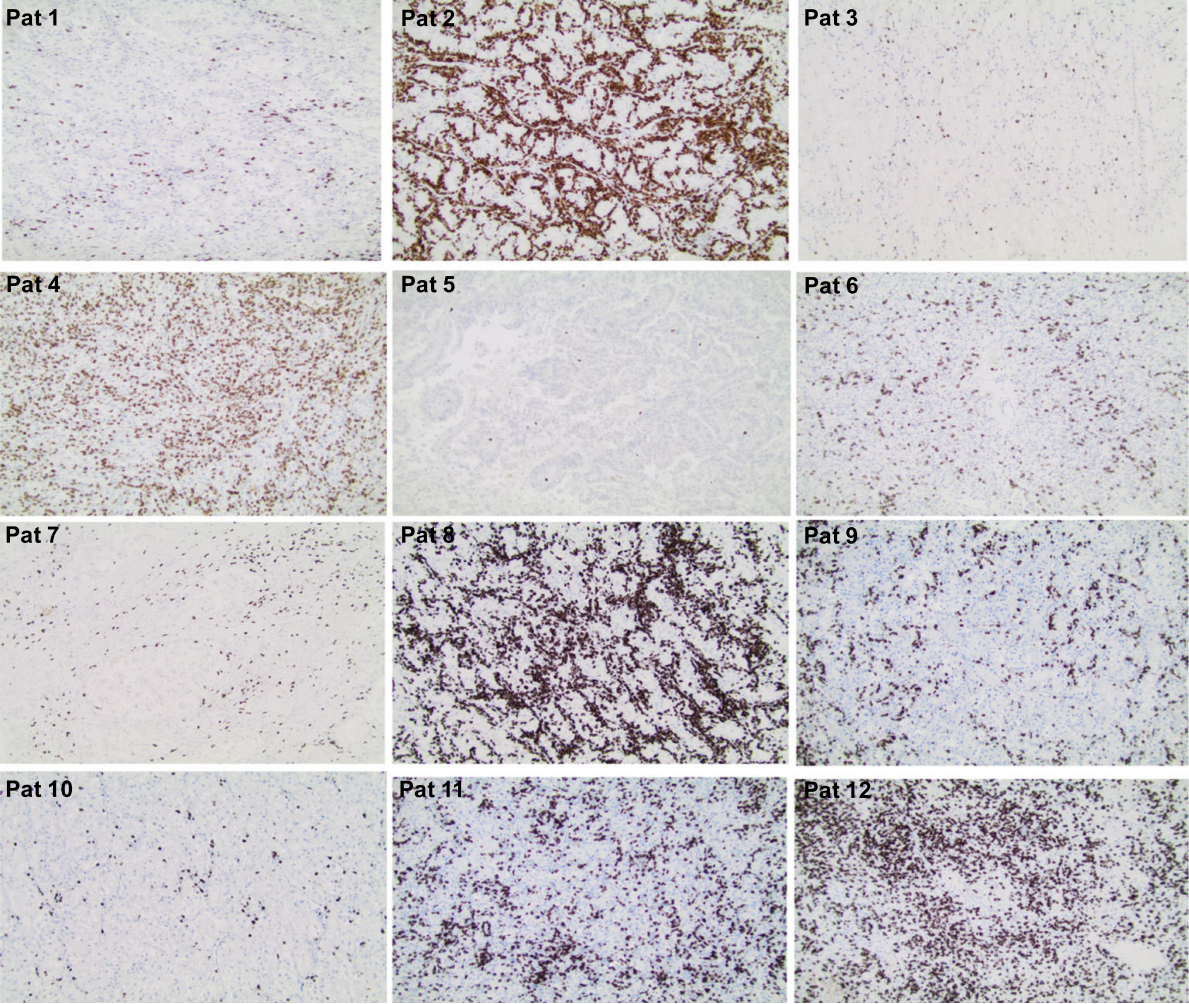
Micrographs illustrating immunohistochemical staining of tissue samples from all 12 surgically removed primary renal RCC tumors with anti-CD8 antibodies. The surgical removal of the tumor-bearing kidney was performed 1–2 weeks after the second (last) administration of ilixadencel. Original magnification × 100. The data in figure originates from Laurell *et al*. [46].

**Fig. 3:**
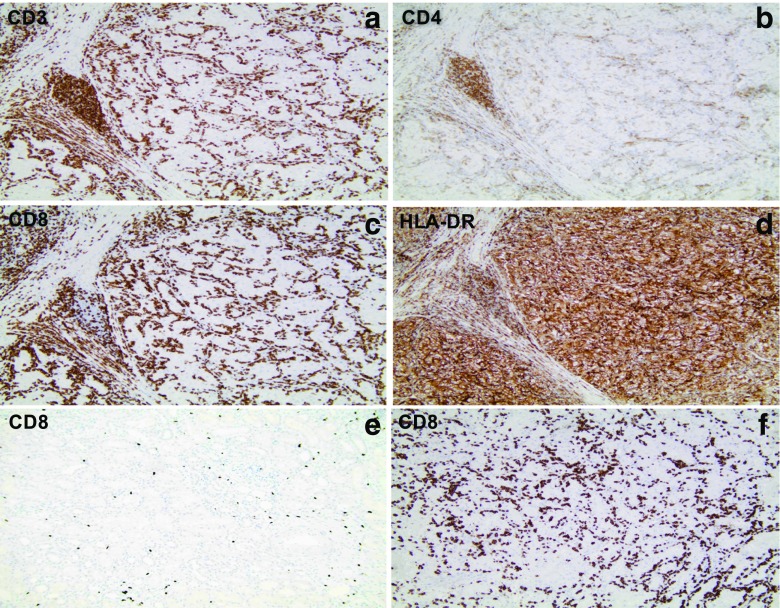
Micrographs illustrating immunohistochemical staining of tissue samples from surgically removed primary renal RCC tumors (surgically removed 1–2 weeks after the second (last) administration of ilixadencel) in consecutive sections from one RCC tumor with high CD8^+^ T cell infiltration (A-D, all from patient 2). (E) illustrates CD8^+^ T cells in normal kidney parenchyma adjacent to tumor areas from patient 2 and (F) illustrates CD8^+^ T cell infiltration in a subcutaneous metastatic lesion from patient 2. Original magnification × 100. The data in figure originates from Laurell *et al*. [46].

Twelve intermediate and poor risk patients with newly diagnosed synchronous metastatic renal cell carcinoma (mRCC) were included in the study (IM-101). A dose of 5–20 × 10^6^ of cells was injected into the renal tumor twice with 2 weeks interval before planned nephrectomy and the patients subsequently received standard of care [46]. No severe adverse events related to ilixadencel were observed. A massive infiltration of CD8^+^ T cells was found in 5 out of 12 removed kidney tumors (see Fig. 2). As illustrated in Fig. 3, the number and infiltration pattern of CD8^+^ T cells were nearly identical to the number and infiltration pattern of CD3^+^ T cells within tumor cell nests and by far outnumbered CD4^+^ T in tumor samples with high intratumoral T cells infiltration. In contrast, CD4^+^ T cells generally predominated in the stroma surrounding tumor cell nests (Fig. 3b). The normal kidney tissue areas adjacent to tumor areas, including those adjacent to tumor areas with high CD8^+^ T cell infiltration, were generally only weakly infiltrated with CD8^+^ T cells (Fig. 3e). In the only evaluable metastatic lesion surgically removed (subcutaneous lesion removed 1 month after nephrectomy) from a patient with massive CD8^+^ T cell infiltration in the primary kidney tumor, a similar massive infiltration of CD8^+^ T cells was observed (Fig. 3f). Moreover, high CD8^+^ infiltration was generally associated with strong HLA-DR expression in adjacent tumor cells (Fig. 3d).

**Fig. 4:**
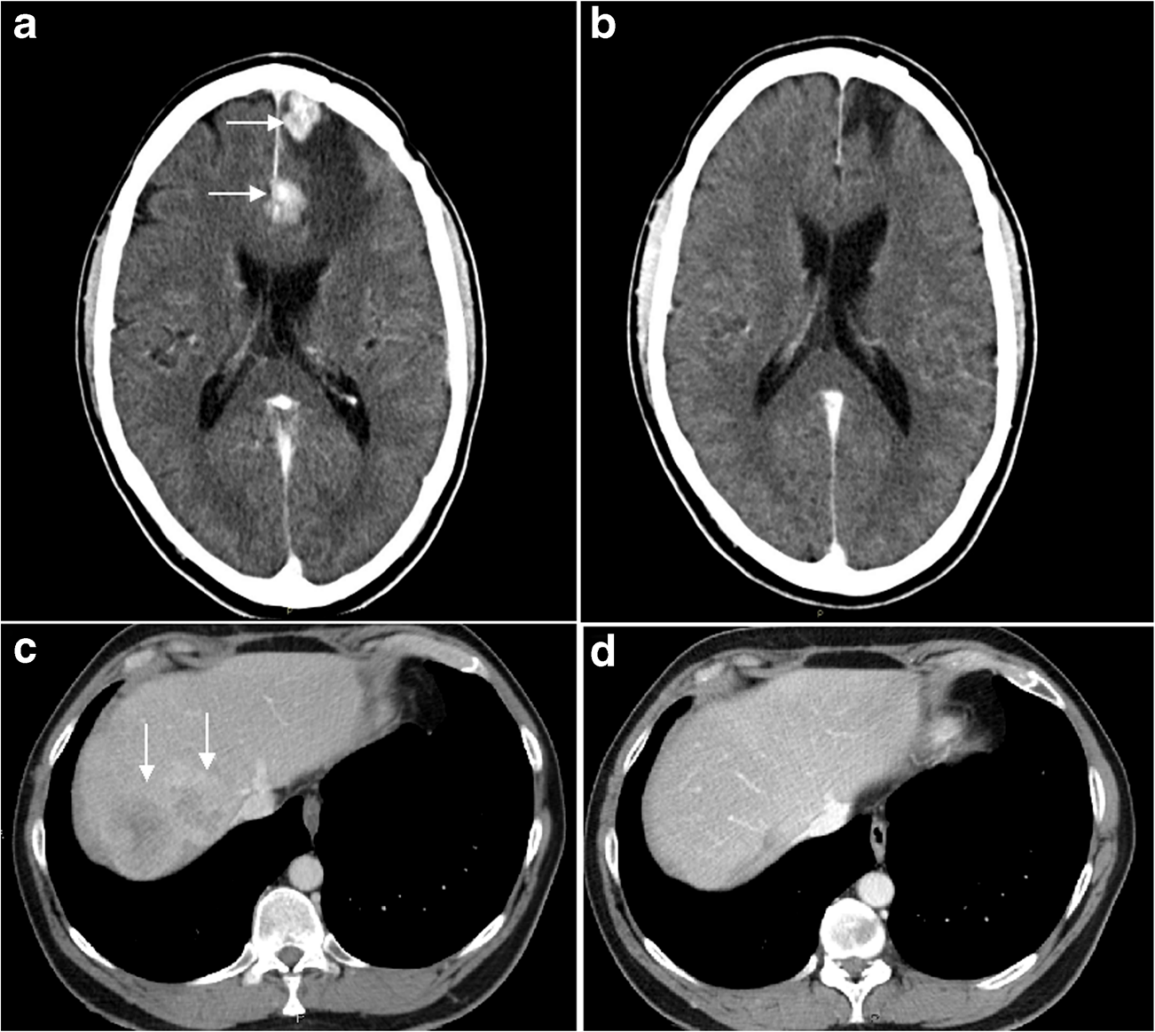
CT-scans from a patient with CNS and liver metastases 4 months after start of ilixadencel treatment but before additional treatment with sunitinib (A and C, respectively) and 6 months (B) or 12 months (D) after start of sunitinib treatment. The data in figure originates from Laurell *et al*. [46].

No objective tumor response was observed in any patient at 3 months follow up after nephrectomy and 6 out of 11 evaluable patients subsequently received additional treatment with standard tyrosine kinase inhibitors (TKI) at progression. Three of these 6 patients experienced an objective tumor response, including one sunitinib-treated patient, showing strong intratumoral infiltration of CD8^+^ T cells in the removed tumor, who responded with a complete and durable regression (> 52 months, still ongoing) of 4 brain metastases (and also all liver metastases) (Fig. 4). This highly favourable long-lasting response was unexpected since mRCC-derived CNS metastases are not expected to respond to sunitinib treatments [50]; it was hypothesized that the add-on treatment with sunitinib inhibited the tumor-associated immunosuppression by inhibiting MDSCs [7, 51] thereby opening up for a T cell-mediated tumor destruction. Median overall survival (mOS) for the whole patient group was 48.0 months and three patients have passed 5 years survival. These survival data are encouraging in the light of historical data in patients with recently diagnosed (including synchronous) mRCC patients treated with VEGF-targeted therapies, including sunitinib, where a median OS of 15–16 months has been reported [52, 53].

### Phase I Study in Patients with Advanced HCC

This was a single arm, open-label, phase I trial including 18 patients with advanced liver cancer (IM-102), 17 patients with unresectable hepatocellular carcinoma (HCC) and 1 patient with advanced cholangiocarcinoma (CC). Patients were to be treated with three separate injections of ilixadencel (10 or 20 × 10^6^ of cells) directly into their tumor (at approximately day 1, 14 and 42) and patients were followed for 6 months after last injection. The final patient disposition was as follows: 7 patients were treated with ilixadencel as second line systemic treatment, 10 patients were treated as first line treatment of which 6 patients were treated in combination with sorafenib in the first line setting.

There were no life-threatening or fatal treatment-related adverse events (AEs). Overall, with only one exception, all treatment-related AEs were mild-to-moderate. The most common treatment-related AEs were described as fever and/or chills and could be easily managed. The exception was one patient receiving ilixadencel and sorafenib as combination therapy, who presented with a suspected sepsis event (grade 3) that subsequently recovered. As a reference, current standard of care, such as sorafenib or regorafenib, report in the literature [54] and in prescribing information severe (grade 3) drug-related AEs in at least one in three HCC patients treated.

Evidence of systemic immunological response to the treatment, as measured by an increase in α-fetoprotein and/or hTERT specific, IFN-γ producing CD8^+^ T cells in the blood, was demonstrated in 9 out of 13 evaluable patients (69%). Overall survival ranged between 1.6–21.4 months in the total group of 17 HCC patients at closure of study with three patients still alive. More in-depth group and patient analyses are ongoing.

The patient with advanced cholangiocarcinoma was included by a study amendment and received three intratumoral administrations of ilixadencel, each dose administered the day after transarterial chemo-embolization (TACE) with doxorubicin-eluting microspheres, aimed at inducing immunogenic tumor-cell death within the ilixadencel-treated tumor lesion. The frequency of IFN-γ producing peripheral blood CD8^+^ T cells against hTERT and AFP was increased 1 week after the third dose of ilixadencel when compared to baseline levels. The patient’s tumor showed a partial response at 3 months. At 6 months the tumor progressed and standard gemcitabine/cisplatine (G/C) regimen was started, which resulted in a nearly complete tumor response. Overall, this patient survived for 41 months post enrolment. These survival data are encouraging when comparing to historical data for patients with advanced CC on standard G/C treatment with an expected overall survival of 12 months [55]. We therefore hypothesize that combination of ilixadencel-induced recruitment and activation of endogenous DCs, local doxorubicin-induced enhancement of immunogenic cell death as well as the addition of gemcitabine that is known to deplete MDSCs [7, 56] may act in synergy as indicated by objective tumor response and long survival.

### Phase I Study in Patients with Gastrointestinal Stromal Tumors

This study is a prospective single arm, open-label phase I safety study of ilixadencel in patients with progressing gastrointestinal stromal tumors (GIST) (IM-103) during ongoing second, third or fourth line treatment with tyrosine kinase inhibition therapy. A total of 6 patients have been enrolled; the analysis of primary outcome measures of safety and tolerability, as well as initial secondary outcomes on efficacy, tumor response and progression-free survival, are expected in the course of 2019.

### Phase II Study IM-201 (MERECA) with Ilixadencel Pre-Nephrectomy Followed by Sunitinib Post-Nephrectomy Compared with Standard Treatment with Sunitinib Monotherapy Post-Nephrectomy

Based on data from the initial phase I trial in patients with newly diagnosed mRCC, suggesting that ilixadencel treatment followed by standard sunitinib treatment may act synergistically, a phase II mRCC study was initiated. This study is an open-label, randomized study comparing ilixadencel pre-nephrectomy, followed by sunitinib treatment with only sunitinib post-nephrectomy. Patient recruitment has been completed and a total of 88 newly diagnosed mRCC patients have been randomized into the trial conducted at 28 centers in eight European countries and the US. The 18-month follow-up period for the last patient treated will be completed around mid-year 2019.

### Planned Phase Ib/II Study Multi-Indication Trial IM-202 Including Head and Neck, Lung and Gastric Cancer in Combination with a PD-1/PD-L1 Inhibitor

Based on the potential synergy observed between ilixadencel and drugs that inhibit immunosuppression (sunitinib and gemcitabine), including unpublished preclinical data indicating that ilixadencel and anti-PD-1 treatment have a synergistic anti-tumor effect in the CT-26 mouse tumor model, a new combination study is planned: this is a randomized, open-label, multicenter, phase Ib/II trial evaluating the safety and efficacy of intratumorally-administered ilixadencel in combination with checkpoint inhibitor (CPI) in advanced cancer subjects who are candidates for CPI therapy. The indications will be advanced (unresectable or metastatic) head and neck squamous-cell carcinoma of the (HNSCC), non-small cell lung cancer (NSCLC), and gastric or gastroesophageal junction (GEJ) adenocarcinoma.

## Summary

The cross-presenting function of DCs is essential for the therapeutic effect of CPIs, but the baseline CTL-priming function is suboptimal. These observations suggest the potential to devise intratumoral (*in situ*) administration of immune primers to enhance cross-presentation of cell-associated tumor antigens, including neoantigens, and thereby increase the efficacy of CPIs. Based on our preclinical and early clinical findings, the intratumoral administration of ilixadencel in the clinical setting is expected to induce a cascade of immunological events, including recruitment of NK cells, DCs and T cells that ultimately leading to desirable Th1-polarized activation of DCs and efficient MHC class I cross-presentation of cell-associated tumor antigens. Clinical data are further suggesting that the combination of ilixadencel and drugs reducing tumor-derived immunosuppression may have a synergistic antitumor effect.

### Acknowledgments and Disclosures

AKP, JK, EH, SZ, SL and PS report ownership of stocks in Immunicum AB and are all Immunicum employees. MJ report ownership of stocks in Immunicum AB and is a consultant to Immunicum AB.

### Data Availability

Data sharing not applicable to this article as no datasets were generated or analysed during the current study.

## Abbreviations

ATMP: Advanced Therapy Medicinal Product
cDC1: Conventional type 1 DCs
CPI: Check Point Inhibitors
CTL: Cytotoxic T Lymphocytes
DC: Dendritic Cell
HLA: Human Leukocyte Antigen
IFN: Interferon
IL: Interleukin
INN: International Nonproprietary Name
MDSC: Myeloid-Derived Suppressor Cells
MoDC: Monocyte-derived Dendritic Cell
NK: Natural Killer
PBMC: Peripheral Blood Mononuclear Cells
PD-1: Programmed Cell Death Protein-1
PD-L1: Programmed Cell Death-Ligand 1
TAP: Transporter associated with Antigen Processing
TLR: Toll-Like Receptor
TME: Tumor Microenvironment
TNF: Tumor Necrosis Factor
